# Avanço do Transplante Cardíaco no Brasil: É Hora de se Construir um Banco de Dados Nacional?

**DOI:** 10.36660/abc.20210104

**Published:** 2021-04-08

**Affiliations:** 1 Universidade de São Paulo Faculdade de Medicina Hospital das Clínicas São PauloSP Brasil Universidade de São Paulo Faculdade de Medicina Hospital das Clínicas Instituto do Coração, São Paulo, SP – Brasil.

**Keywords:** Insuficiência Cardíaca/cirurgia, Transplante Cardíaco/tendências, Imunossupressão/complicações, Brasil

O transplante cardíaco (TC) é o tratamento de escolha para insuficiência cardíaca grave.^1^ A sobrevida melhorou substancialmente desde o primeiro transplante há 50 anos, principalmente após a introdução de inibidores de calcineurina e melhor manejo das complicações relacionadas à imunossupressão.^2^ Na América Latina, o Brasil é reconhecido pelo elevado número de TCs realizados anualmente.^3^ Apesar de sua importância, existem poucos estudos sobre sobrevida, imunossupressão e complicações do TC no Brasil.

Um estudo retrospectivo de coorte aberta conduzido no Brasil é apresentado nesta edição dos Arquivos Brasileiros de Cardiologia.^4^ O artigo mostra dados importantes sobre a epidemiologia, sobrevida e complicações em receptores de TC entre os anos de 2000 e 2015. A sobrevida mediana nessa coorte foi de 8,3 anos, e a taxa de sobrevida de um ano e de cinco anos foi de 70,9% e 59,5%, respectivamente. Esses resultados são melhores aos comparados aos dados do período entre 1984 e 1999 no Brasil, quando as taxas de sobrevida de um ano e de seis anos eram de 66% e de 54%, respectivamente,^5^ sugerindo melhora no cuidado pós-transplante. No entanto, essa sobrevida é inferior aos 12,2 anos de sobrevida e às taxas de sobrevida de um ano (81%) e de 5 anos (69%) relatadas pela Sociedade Internacional de Transplante de Coração e Pulmão (ISHL, International Society of Heart and Lung),^6^ provavelmente devido a diferenças sociodemográficas e econômicas entre o Brasil e países desenvolvidos.

A fim de entender os principais fatores associados com as taxas de sobrevida no Brasil, os autores estudaram diferentes variáveis e regiões geográficas do Brasil. Os autores encontraram que uma idade mais avançada do receptor [HR 1,014 (IC 95%: 1,004-1,025), p=0,006], sul do Brasil como local onde o TC foi realizado [HR: 1,592 (IC 95%: 1,240-2.044), p<0,001], e infecção pós-transplante [HR: 1,912 (IC 95%: 1,136-3,243), p=0,015] foram fatores de risco significativos para perda do enxerto. Quanto aos regimes de imunossupressão, drogas antiproliferativas foram associadas com menor mortalidade, enquanto os inibidores de calcineurina não tiveram impacto sobre a sobrevida após o TC.

Pelo fato de os dados terem sido extraídos de banco de dados administrativos, algumas informações faltantes afetaram os resultados: a etiologia da insuficiência cardíaca não estava clara em 69.1% dos casos; o uso de corticosteroides não estava descrito; as causas de morte também não foram relatadas, e nenhum dado sobre rejeição do enxerto ou doença vascular do enxerto foi relatado. Todas essas variáveis são diretamente relacionadas a melhorias no tratamento e sobrevida do TC.^6^^,^^7^ Ainda, no registro da ISHLT de 2017, mais de 30% das mortes dos recipientes de TC em todo o mundo, no primeiro ano pós-transplante, foram causadas por doenças infecciosas,^6^ ao passo que nesta coorte, somente 3,7% dos pacientes tinham registro de infecções. De fato, a principal causa de morte no primeiro ano pós-transplante segundo a ISHLT é infecção.^8^

Essas disparidades nos dados devem-se provavelmente à natureza retrospectiva da pesquisa. Algumas questões continuam sem respostas, e talvez um banco de dados nacional unificado ajudaria a preencher essas lacunas na literatura brasileira. Apesar dessas limitações, esta publicação certamente amplia o conhecimento sobre o cenário do TC no Brasil, uma vez que esse é o único estudo coorte recente que correlaciona sobrevida, imunossupressão, e variáveis clínicas. O estudo também destaca pontos importantes tais como diferenças regionais, problemas do sistema público de saúde, e melhora na sobrevida do TC nas últimas décadas no Brasil.
