# Supplementary material for: Avanço do Transplante Cardíaco no Brasil: É Hora de se Construir um Banco de Dados Nacional?
Source: Arq Bras Cardiol. 2021 Apr 8;116(4):754–5. [Article in Portuguese] doi: 10.36660/abc.20210104 (PMC8121400; doi:10.36660/abc.20210104)
Supplement: Translation

# Translation: Progress in Heart Transplantation in Brazil: is it Time to Build a National Database?

**Affiliations:** 1 São PauloSP Brazil Universidade de São Paulo Faculdade de Medicina Hospital das Clínicas Instituto do Coração, São Paulo, SP – Brazil.

**Keywords:** Heart Failure/surgery, Heart Transplantation/trends, Survival, Immunosupression/complications, Brazil

Heart transplantation (HT) is the therapy of choice for advanced heart failure.^1^ Survival has greatly improved since the first heart transplant 50 years ago, especially after the introduction of calcineurin inhibitors and better management of complications related to immunosuppression.^2^ In Latin America, Brazil is renowned for the high number of HTs performed annually.^3^ Despite its importance, reports on survival, immunosuppression and complications of HT are scarce in Brazil.

A retrospective open cohort of HT in Brazil is presented in this issue of Arquivos Brasileiros de Cardiologia.^4^ The article shows important data regarding epidemiology, survival and complications of HT recipients between 2000 and 2015. Median survival in this cohort was 8.3 years, and one- and 5-year survival rates were 70.9% and 59.5%, respectively. These results are better than those from 1984 to 1999, when the one- and 6-year survival rates were 66% and 54% respectively,^5^ suggesting improvement in post-transplant care. However, this median survival is lower than 12.2-year survival and one- and 5-year survival of 81% and 69% reported by the International Society of Heart and Lung Transplantation (ISHLT),^6^ probably due to sociodemographic and economic differences between Brazil and developed countries.

In order to understand the main factors associated with survival rates in Brazil, the authors studied different variables and geographic regions of Brazil. They found that the recipient's older age (HR 1.014 [95%CI: 1.004-1.025], p=0.006), South of Brazil as the location where HT was performed (HR: 1.592 [95%CI: 1.240-2.044], p<0.001), and post-transplant infection (HR: 1.912 [IC 95%: 1.136-3.243], p=0.015) as significant risk factors for graft loss (death or retransplant). Regarding immunosuppressive regimens, antiproliferative drugs were associated with lower mortality, while calcineurin inhibitors showed no impact on survival after HT.

As the data were extracted from three administrative databases, some missing information affected the results: the etiology of heart failure was not clear in 69.1% of cases; the use of corticosteroids was not described; causes of death were also not reported and no data on graft rejection or cardiac allograft vasculopathy were reported. All of these variables are directly related to improvements in HT treatment and survival.^6^^,^^7^ Also, in the ISHLT 2017 registry, more than 30% of HT recipients deaths worldwide in the first year post-transplant were caused by infectious diseases,^6^ while in this cohort only 3.7% of all patients had infections registered. Indeed, infection is the main cause of death in the first year pos-transplant according to the ISHLT.^8^

Such disparities in the data are probably due to the retrospective nature of the research. Some questions remain unanswered, and perhaps a unified national database would help to fulfill these gaps in Brazilian literature. Despite these limitations, this publication certainly increases the knowledge about HT scenario in Brazil, since this is the only recent cohort study correlating survival, immunosuppression, and clinical variables. It also highlights important points such as regional differences, public health problems, and improvement in HT survival in the last decades in Brazil.
